# Validation of the Chinese Version of the Stigma Scale of Epilepsy

**DOI:** 10.3389/fneur.2022.796296

**Published:** 2022-02-07

**Authors:** Yuanxia Wu, Kailing Huang, Shirui Wen, Bo Xiao, Li Feng

**Affiliations:** ^1^Department of Neurology, Xiangya Hospital, Central South University, Changsha, China; ^2^National Clinical Research Center for Geriatric Disorders, Xiangya Hospital, Central South University, Changsha, China

**Keywords:** epilepsy, the stigma scale of epilepsy, validation, China, stigma

## Abstract

**Purpose:**

This study was carried out to test the validity and reliability of the Chinese version of the Stigma Scale of Epilepsy (SSE), with aim to better understand the public stigmatizing attitudes of epilepsy in China and help elucidate stigma determinants for interventions.

**Methods:**

The SSE was translated into Simplified Chinese Mandarin. In this study, most of the participants were enrolled *via* convenience sampling by randomly distributing questionnaires on the streets and parts of the participants were recruited by an online platform named Wenjuanxing. We assessed the psychometric properties of the SSE in 310 Chinese native-speaker. Cronbach's alpha was tested for reliability. Index of Content Validity (CVI) was calculated. Exploratory and confirmatory analysis were used to explore the factor structure and verify the validity of SSE.

**Results:**

The Cronbach's alpha is 0.936 for the overall scale, and the CVI value is greater than 0.78. The exploratory factor analysis (EFA) extracted SSE six factors: the fear of seizure attacks (factor 1), sympathy for patients with epilepsy (PWEs) (factor 2); difficulties faced by PWEs (factor 3); speculation on PWEs' feeling (factor 4); discrimination against PWEs (factor 5); and knowledge about epilepsy (factor 6). The item 13 was proven to be problematic and has been eliminated. The confirmatory factor analysis (CFA) ensured the great construct validity (χ^2^/SD = 1.725, goodness of fit index (GFI) = 0.916, and root mean square error of approximation (RMSEA) = 0.048), convergent validity (the factor loads of each item corresponding to each latent variable >0.6, average variance extracted (AVE) > 0.5, and composite reliability (CR) > 0.7), and discrimination validity (all of the absolute value of correlation coefficient are <0.5,and less than the square root of AVE) of the SSE.

**Conclusions:**

The Chinese version of the SSE scale was a valid and reliable tool to measure epilepsy-associated stigma in the Chinese society.

## Introduction

Epilepsy is a chronic neurological disease characterized as excessive hyper synchronized discharge of brain neurons (1, 2). Patients with epilepsy (PWEs) not only suffer from the physical problems (such as, fractures and bruising from injuries related to seizures), but also a series of complex psychosocial disorders, such as anxiety, depression, suicidal risk, stigma and discrimination, exclusion, and overprotection (1, 3–6). Among these psychosocial disorders, stigma is the one with great impacts on the recovery, prognosis, and quality of life among PWEs (7, 8), which is even greater than the adverse reactions of epileptic seizure and antiepileptic drugs (9).

The stigma related to epilepsy prevails globally (10), and it may be much more serious in China than elsewhere. To date, there are more than 9 million PWEs in China with a treatment gap of 63% (10–12). The incidence of stigma among PWEs in urban and rural areas are 71% and 89%, respectively (13). Guo pointed out that the sources of PWEs' stigma in China come from three main aspects: seizure attacks, social misconceptions, and negative attitudes toward epilepsy and negative psychological factors of patients (14). Unlike other chronic diseases, such as diabetes, the onset of seizures is sudden, uncontrollable, and always accompanied with a series of unsightly manifestations, such as sudden falls, limbs twitching, and foaming at the mouth (15), which may be a shock or an unacceptable thing to the witnesses for the first time. These feelings will alienate the witnesses from PWEs, which is a form of stigma. Besides, social misconception about epilepsy may contribute a lot to epilepsy-associated stigma (16). In rural areas of China, it is believed that epilepsy was caused by seeing ghosts, being possessed by devils, and was always thought to be a mental disease rather than neurological disease, with high possibility to be passed on to the next generation (17, 18). About 14% of parents do not want their children to play with children with epilepsy, and 75% of parents do not want their children to marry a PWE (19). Even the medical staffs in basic-level hospitals from southern China, they still showed negative and conservative attitudes toward PWEs (20). For PWEs themselves, they feel that they are physically defective and worry about their future career and social life because of their misperceptions about epilepsy, which will definitely lead to the aggravation of felt stigma (21). Therefore, it is of great importance to make everyone aware of the problems faced by PWEs and fundamentally change misconceptions of people and explore stigma determinants for social interventions in China.

To achieve effective stigma reducing interventions, stigma need to be measured accurately. The Epilepsy Stigma Scale (EES) and the Kilifi Stigma Scale of Epilepsy (KSSE) are the commonly used scales for evaluating self-stigma of PWEs (22, 23). From the perspective of people without epilepsy, the Stigma Scale of Epilepsy (SSE), developed by Brazilian researchers, has been widely applied all over the world, such as Zambia, the Czech Republic, Italy, India, and Korea (24–31). Some studies used “Public Attitudes Toward Epilepsy” scale (PATE) to evaluate the epilepsy-associated knowledge, attitudes, and practices (KAP) among the public (32, 33). But this scale lacks the focus on emotional reaction of public to epilepsy and PWEs. It is known that the PWEs are always accompanied with mood disorder, which would have effect on stigma (34). Furthermore, the public emotional reactions toward epilepsy or seizure attacks may be a source of stigma of PWEs (29). Thus, in our study, we chose the SSE to assess the epilepsy-related stigma and related emotional reaction from the perspective of people without epilepsy. Considering that its high internal consistency and validity has not been confirmed in China, we proposed to investigate the psychometric properties of the SSE and validate the Chinese version of SSE to access the epilepsy-related stigmatization of the public in China.

## Methods

### Subjects

Chinese-native speakers were enrolled *via* convenience sampling by randomly distributing questionnaires on the streets of Changsha, China, and parts of the participants were recruited by an online platform named Wenjuanxing. The participants in the study had no history of seizures or epilepsy. No other exclusion criteria were made. Written consents were obtained and all questionnaires were administered anonymously. The sociodemographic characteristics of participants, such as age, gender, marital status, educational levels, medical background, and family history of epilepsy were recorded.

### Ethics Statement

Our study has been approved by the Ethics Committee of the Xiangya Hospital of Central South University [No. 201912528]. All the participants were informed about the purpose and significance of our study and that their answers to the scale will be used in our scientific research. All the subjects provided informed consent in writing.

### Measurements

The SSE scale is developed and validated by Fernandes (24, 25, 29). The SSE scale is a five-dimensional 24-item scale that measures public stigma toward epilepsy. The first dimension includes one item for the understanding of disease essence. The second dimension consists of four items about the emotions when the people witness a seizure. The third and fourth dimension both have seven items, reflecting the difficulties face by PWEs and the public emotions from the patient's point of view, respectively. The fifth dimension contains 5 items showing the prejudice and discrimination associated with epilepsy. Each item has four response categories with 1 being “agree not at all,” 2 being “a little,” 3 being “a lot,” and 4 being “totally agree.” The internal consistency of the SSE for the score showed a general Cronbach's coefficient of 0.88 for the PWEs and 0.81 for people in the community.

### Translation of the SSE Into Simplified Mandarin Chinese

To be consistent with rules for the translation of research tools and make the questionnaire acceptable and comprehensible, the original SSE scale was translated into Mandarin Chinese by the two separate and bilingual researchers, who have no medical knowledge and systematic training. Then, the authors modified the translated words because of conservative culture and thinking. A team of clinical neurologists and psychologists checked and determined the better version. Furthermore, the better Chinese version of SSE was translated back into English and compared with the original scale. Modifications are made until the acceptable Chinese version is agreed. After reviewing the logic and typo errors, twenty native Chinese speakers from different background were asked to complete the draft and point out any ambiguities or difficulties in understanding. All the revisions were contained in the last version of the scale. After that, we invited another twenty native Chinese speakers, and they did not raise any new questions. Therefore, the last version was finalized.

### Sample Size

The sample was calculated according to the widely quoted rule of thumb (35), in which 10 or 5 subjects at a minimum are required for every item being analyzed. Not <120 or 240 subjects are required in our research. As Comrey (36) suggested that “a sample size of 100 is poor, 200, fair; 300, good; 500, very good; and 1,000, excellent, we included 300 subjects at least in our study.”

### Data Analysis

Data were input with excel and analyzed with IBM SPSS Statistics 25 software and AMOS 24 software. Descriptive statistics were used to characterize the subjects and distribution of SSE scores. Categorical variables were expressed as number and percentage.

#### Reliability

The internal consistency of the scale was assessed by corrected item-total correlations, Cronbach's alpha and Cronbach's Alpha if item removed. The value of corrected item-total correlations should be >0.4. An alpha value of 0.7–0.9 is acceptable, >0.9 is ideal.

#### Validity

We invited six experts from the psychology, epidemiology, and neurology departments to evaluate the content validity of the SSE. Experts were asked to make a choice on how relevant (or representative) each item was to the corresponding content dimension, and to suggest items that needed to be added or adjusted. In general, the options are 4-point rating: 1 = inappropriate, 2 = relatively inappropriate, 3 = relatively appropriate, and 4 = appropriate. The higher the score, the more appropriate the item. Index of Content Validity (CVI) was calculated to evaluate to the relevance of the item to the corresponding content dimension, and the value of CVI should be not <0.78. Before exploratory factor analysis (EFA), The Kaiser–Meyer–Olkin (KMO) test was applied for sampling adequacy and whether the data met the criteria of principal component analysis (PCA). The KMO value should be >0.6 and the factor loading coefficient of the item on the corresponding factor need to be >0.5. We used the varimax rotation and scree plot for EFA. Eigenvalue >1 was used to identify the number of extracting factors in the scree plot. Confirmatory factor analysis (CFA) was performed to further check the factor structure of the translated Chinese version of SSE. Average variance extracted (AVE) and composite reliability (CR) are commonly used indicators of polymerization validity. In general, AVE is >0.5 and CR value is >0.7 (37), indicating high polymerization validity. The discriminant validity can be tested by comparing the AVE square root with the correlation value. If the AVE square root is greater than the correlation value, the discriminant validity is good. We performed a comparison between the total score of SSE and scores of each factor with medical background and family history of epilepsy using Mann–Whitney test to prove the validity.

## Results

### The Chinese SSE

After the standard translation process described above, the finalized Chinese version of the SSE was created (Appendix 1). The average time to fill out the scale was about 5 min.

### Demographic Data

In total, 310 subjects completed the questionnaire. The ages of the 310 respondents in the study varied between 15 and 74 years, with 57.7% male respondents, 40.6% married, 98.7% having a minimum education of 9 years, 21.0% having medical background, and 3.2% having family history of epilepsy (Table 1). Table 2 depicts the percentage distributions of the responses to each item on the Chinese SSE scale.

**Table 1:** Demographic characteristic of participants (*n* = 310).

		**Number (%)**
Sex	Male	179 (57.7)
	Female	131 (42.3)
Age	under 18 years old	7 (2.3)
	18–60 years old	296 (95.4)
	60 years old or above	7 (2.3)
Marital status	Married	126 (40.6)
	Single	178 (57.4)
	Divorced	6 (1.9)
Education level	Primary or under	4 (1.3)
	Middle or High School	106 (34.2)
	College or above	200 (64.5)
Medical background	Yes	65 (21.0)
	No	245 (79.0)
Family history of epilepsy	Yes	10 (3.2)
	No	300 (96.8)

**Table 2:** Felt stigma measures using the Stigma Scale of Epilepsy (SSE).

**Stigma scale of epilepsy, number (%)**				
	**Not at all**	**A little**	**A lot**	**Totally**
Question 1: Do you think that people with epilepsy feel able to control their own epilepsy?				
1. Control	190 (61.3)	87 (28.1)	28 (9.0)	5 (1.6)
Question 2: How would you feel when you see an epileptic seizure?				
2. Scared	57 (18.4)	151 (48.7)	50 (16.1)	52 (16.8)
3. Fear	75 (24.2)	129 (41.6)	56 (18.1)	50 (16.1)
4. Sadness	22 (7.1)	82 (26.5)	80 (25.8)	126 (40.6)
5. Pity	15 (4.8)	58 (18.7)	61 (19.7)	176 (56.8)
Question 3: Which difficulties do you think people with epilepsy have in their daily lives?				
6. Relationships	19 (6.1)	117 (37.7)	71 (22.9)	103 (33.2)
7.Work	12 (3.9)	104 (33.5)	74 (23.9)	120 (38.7)
8. School	36 (11.6)	107 (34.5)	73 (23.5)	94 (30.3)
9. Friendships	27 (8.7)	113 (36.5)	72 (23.2)	98 (31.6)
10. Sexual	40 (12.9)	131 (42.3)	73 (23.5)	66 (21.3)
11. Emotional	34 (11.0)	126 (40.6)	81 (26.1)	69 (22.3)
12. Prejudice	22 (7.1)	116 (37.4)	91 (29.4)	81 (26.1)
Question 4: How do you think that people with epilepsy feel?				
13. Worried	15 (4.8)	92 (29.7)	105 (33.9)	98 (31.6)
14. Dependent	31 (10.0)	170 (54.8)	71 (22.9)	38 (12.3)
15. Incapable	42 (13.5)	148 (47.7)	78 (25.2)	42 (13.5)
16. Fearful	25 (8.1)	118 (38.1)	94 (30.3)	73 (23.5)
17. Depressed	22 (7.1)	136 (43.9)	89 (28.7)	63 (20.3)
18. Ashamed	18 (5.8)	134 (43.2)	99 (31.9)	59 (19.0)
19. The same as those without epilepsy	54 (17.4)	147 (47.4)	61 (19.7)	48 (15.5)
Question 5: In your opinion, the prejudice in epilepsy will be related to?				
20. Relationships	33 (10.6)	129 (41.6)	84 (27.1)	64 (20.6)
21. Marriage	26 (8.4)	112 (36.1)	97 (31.3)	75 (24.2)
22. Work	23 (7.4)	120 (38.7)	94 (30.3)	73 (23.5)
23. School	33 (10.6)	124 (40.0)	90 (29.0)	63 (20.3)
24. Family	78 (25.2)	119 (38.4)	72 (23.2)	41 (13.2)

### Validity

**Figure 1:**
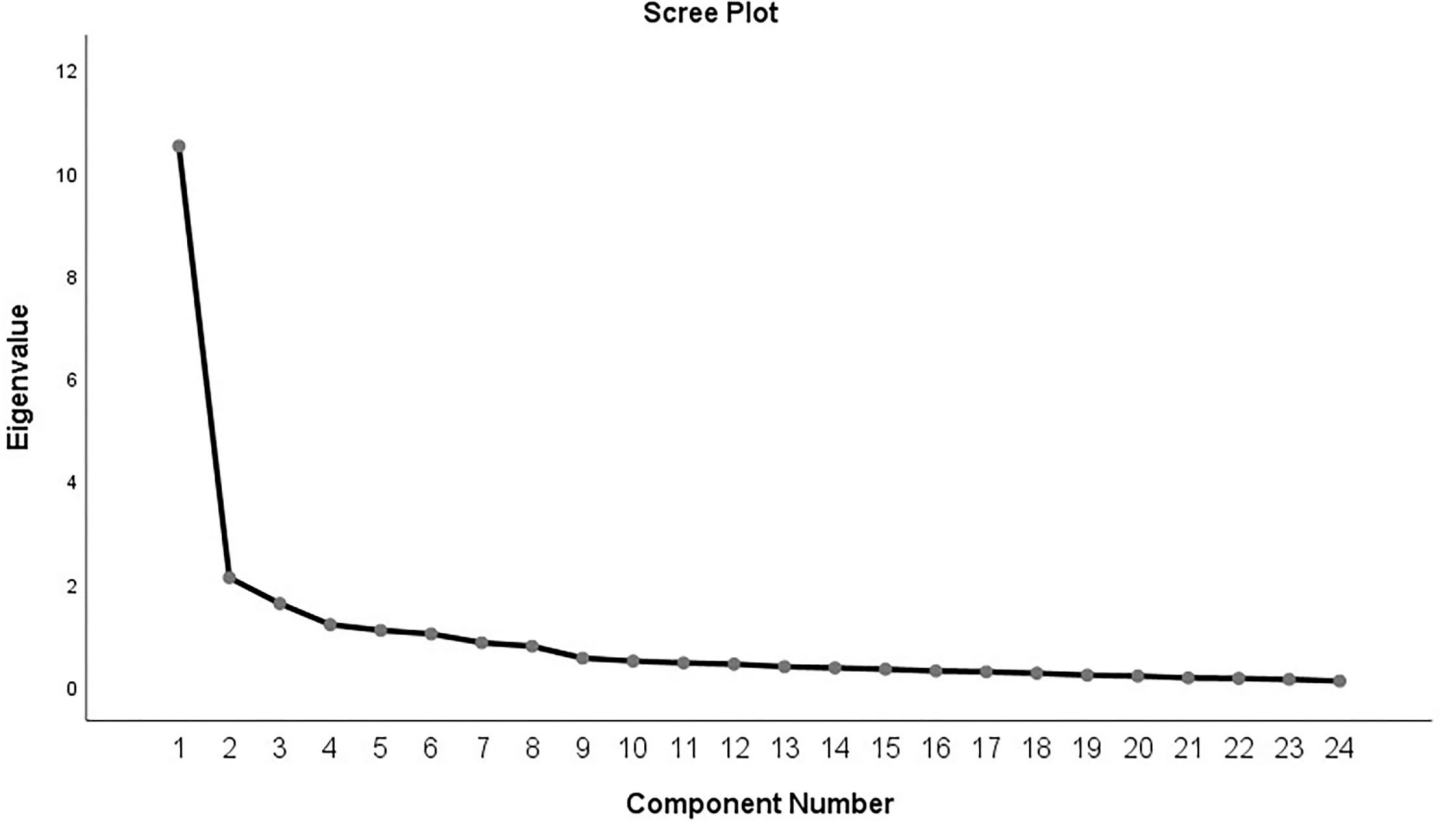
The scree plot of the items related to stigma toward epilepsy in normal people. The scree plot was acquired by principal component analysis (PCA) with correlation matrix.

The KMO test and Bartlett's test of sphericity were performed before PCA. The KMO value was 0.913, and Bartlett's test value was 5,226.218 (*p* = 0.001), suggesting that the data are suitable for a factor analysis. Researchers conducted EFA under a condition of undefined factor number. Six factors (eigenvalue >1) were extracted. The cumulative variance contribution rate (%) of six factors was 73.076%. Moreover, the scree plot indicated that the 6-factor structure was suitable for the scale (Figure 1). The results of rotated component matrix was shown in Table 3. The bold vale of Table 3 means the factor loading of the item in the corresponding component is >0.5, which indicates the item should be attributed to this component. Based on above theoretical analyses, the interpretation of factors given in Table 3 was recommended as follows: factor 1 refers to the public's fear of seizure attacks; factor 2 refers to the public's sympathy for PWEs; factor 3 refers to the difficulties faced by PWEs; factor 4 refers to the public's speculation on PWEs' feeling; factor 5 refers to the discrimination against PWEs; and factor 6 refers to the knowledge about epilepsy. However, the varimax rotation indicated that the factor 6 only has one item. Thus, the item 1 was not included in the model and a five-factor structure was extracted. Besides, item 13 had a factor loading (>0.5) both in the factor 3 (difficulties faced by PWEs) and factor 4 (speculation on PWEs' feeling), which means item 13 is not a specifically targeted question to assess the public's speculation on PWEs' feeling. It may also, to some extent, reflect the difficulties faced by PWEs in the Chinese culture. This would bring errors and biases to subsequent analysis, thus. The item 13 was excluded. As for the Q2, although its item-total correlations are relatively low than other questions, we did not exclude it because the Cronbach's α values were close to the total Cronbach's α value no matter which item in Q2 was deleted, as shown in Table 4. Based on the aforementioned analyses, the final version of the scale consisted of 5 factors and 22 items.

**Table 3:** Rotated component matrix.

	**Component**					
	**1**	**2**	**3**	**4**	**5**	**6**
Item 1	−0.042	0.024	0.014	−0.056	0.038	**0.962**
Item 2	**0.925**	0.138	0.036	0.053	0.036	−0.017
Item 3	**0.924**.	0.147	0.101	0.108	0.001	−0.028
Item 4	0.056	**0.872**	0.125	0.095	0.129	−0.04
Item 5	0.314	**0.797**	0.151	0.138	0.052	0.081
Item 6	−0.013	0.144	**0.758**	0.192	0.304	0.014
Item 7	0.02	0.195	**0.806**	0.155	0.248	−0.035
Item 8	0.048	0.042	**0.801**	0.228	0.289	−0.077
Item 9	0.023	0.113	**0.788**	0.23	0.271	−0.084
Item 10	0.077	0.069	**0.741**	0.248	0.191	0.035
Item 11	0.094	0.004	**0.664**	0.303	0.173	0.095
Item 12	0.103	−0.052	**0.611**	0.381	0.174	0.16
Item 13	−0.006	0.203	**0.503**	**0.591**	−0.019	0.002
Item 14	0.107	−0.02	0.294	**0.682**	0.227	0.175
Item 15	0.015	0.043	0.236	**0.72**	0.37	−0.004
Item 16	0.044	0.124	0.286	**0.736**	0.251	−0.069
Item 17	0.072	0.008	0.295	**0.773**	0.252	−0.111
Item 18	0.06	0.084	0.307	**0.808**	0.266	−0.055
Item 19	0.055	0.16	0.066	**0.539**	0.28	−0.041
Item 20	−0.017	0.128	0.304	0.306	**0.699**	0.061
Item 21	0.09	0.083	0.294	0.272	**0.76**	−0.095
Item 22	−0.019	0.077	0.35	0.272	**0.769**	−0.032
Item 23	−0.036	0.038	0.335	0.391	**0.689**	0.096
Item 24	0.089	0.013	0.276	0.427	**0.587**	0.163

**Table 4:** Reliability results.

	**Corrected item-total correlation**	**Cronbach's α If item deleted**
Question 1: Do you think that people with epilepsy feel able to control their own epilepsy?		
1. Control	Not included	
Question 2: How would you feel when you see an epileptic seizure?		
2. Scared	0.231	0.939
3. Fear	0.285	0.939
4. Sadness	0.382	0.937
5. Pity	0.341	0.937
Question 3: Which difficulties do you think people with epilepsy have in their daily lives?		
6. Relationships	0.708	0.932
7.Work	0.706	0.932
8. School	0.735	0.931
9. Friendships	0.737	0.931
10. Sexual	0.674	0.932
11. Emotional	0.638	0.933
12. Prejudice	0.636	0.933
Question 4: How do you think that people with epilepsy feel?		
13. Worried	Not included	
14. Dependent	0.65	0.933
15. Incapable	0.704	0.932
16. Fearful	0.698	0.932
17. Depressed	0.709	0.932
18. Ashamed	0.759	0.931
19. The same as those without epilepsy	0.479	0.935
Question 5: In your opinion, the prejudice in epilepsy will be related to?		
20. Relationships	0.672	0.932
21. Marriage	0.693	0.932
22. Work	0.708	0.932
23. School	0.718	0.931
24. Family	0.674	0.932
	Item number	Cronbach's alpha
Factor 1	2	0.887
Factor 2	2	0.737
Factor 3	7	0.920
Factor 4	6	0.897
Factor 5	5	0.900
Total	22	0.936

#### Content Validity

Content Validity was expressed by the CVI. All the I-CVI are >0.78, and the mean CVI value of all items in the scale was 0.97. CVI value of each dimension is 0.83, 0.96, 0.98, 0.98, and 1.00, respectively.

#### Construct Validity

The result of the construct validity is shown in Table 5, all within the range of acceptance or ideal. The CFA produced the following fit indices: **χ^2^**/SD = 1.725, goodness of fit index (GFI) = 0.916, normed fit index (NFI) = 0.937, comparative fit index (CFI) = 0.972, IFI (incremental fit index) = 0.972, Tucker–Lewis index (TLI) = 0.966, and root mean square error of approximation (RMSEA) = 0.048. These excellent findings for all metrics indicate that the results fitted well with the 5-factor construct of the scale.

**Table 5:** Goodness of fit index (GFI).

	**X^**2**^/df**	**RMSEA**	**GFI**	**NFI**	**CFI**	**IFI**	**TLI**
Ideal	≤ 3.0	≤ 0.05	≥0.90	≥0.90	≥0.90	≥0.90	≥0.90
Acceptable	≤ 5.0	≤ 0.10	≥0.80	≥0.80	≥0.80	≥0.80	≥0.80
	1.725	0.048	0.916	0.937	0.972	0.972	0.966

*RMSEA, root mean square error of approximation; GFI, goodness-of-fit index; NFI, normed fit index; CFI, comparative fit index; IFI, incremental fit index; TLI, Tucker–Lewis index*.

#### Convergent Validity

As shown in the Table 6, the factor load of each item corresponding to each latent variable is >0.6,which shows that each item is highly representative of the corresponding latent variable. Besides, the average variance extracted is >0.5, and the composite reliability is >0.7, showing good convergence validity.

**Table 6:** Convergent validity.

			**Estimate**	**AVE**	**CR**
Item 2	< ---	Factor 1	0.812	0.8109	0.8947
Item 3	< ---	Factor 1	0.981		
Item 4	< ---	Factor 2	0.969	0.6513	0.7799
Item 5	< ---	Factor 2	0.603		
Item 6	< ---	Factor 3	0.796	0.6108	0.9155
Item 7	< ---	Factor 3	0.803		
Item 8	< ---	Factor 3	0.886		
Item 9	< ---	Factor 3	0.893		
Item 10	< ---	Factor 3	0.752		
Item 11	< ---	Factor 3	0.647		
Item 12	< ---	Factor 3	0.656		
Item 15	< ---	Factor 4	0.816	0.6142	0.9038
Item 14	< ---	Factor 4	0.725		
Item 16	< ---	Factor 4	0.837		
Item 17	< ---	Factor 4	0.836		
Item 18	< ---	Factor 4	0.872		
Item 19	< ---	Factor 4	0.578		
Item 21	< ---	Factor 5	0.82	0.6721	0.9108
Item 20	< ---	Factor 5	0.776		
Item 22	< ---	Factor 5	0.877		
Item 23	< ---	Factor 5	0.865		
Item 24	< ---	Factor 5	0.754		

*AVE, average variance extracted; CR, composite reliability*.

#### Discriminant Validity

As shown in Table 7, there was significant correlation among factor 1, factor 2, factor 3, factor 4, and factor 5 (*p* < 0.01). All the absolute values of correlation coefficient are <0.5,and less than the square root of AVE. It does not only show the significant correlation, but also the significant degree of distinction between the five factors.

**Table 7:** Discriminant validity.

	**F1**	**F2**	**F3**	**F4**	**F5**
F1	0.8109				
F2	0.243^***^	0.6513			
F3	0.133^**^	0.128^***^	0.6142		
F4	0.149^***^	0.119^***^	0.376^***^	0.6721	
F5	0.087	0.106^**^	0.415^***^	0.423^***^	0.6108
	0.9005	0.807032	0.781537	0.78370913	0.819817

Discriminant validity were significant at *p < 0.05,

** *p < 0.01,*

*** *p < 0.001*.

#### Effect of Medical Background and Family History of Epilepsy

To evaluate the possible effect of medical background and family history of epilepsy on the level of stigmatization, we performed a comparison using Mann–Whitney test for the sum score and scores of each factor. The results are summarized in Supplementary Table 1. It was seen that participants with family history of epilepsy have higher SSE scores than those without family history of epilepsy (*p* = 0.007), as well as three factors out of six factors' scores (factor 2, factor 4, and factor 5). There is statistically no significance of the SSE scores between participants with medical background and participants without (*p* = 0.135), but the scores of factor 1 and factor 2 in participants without medical background are higher than those with medical background (*p* < 0.001 and *p* = 0.002, respectively).

### Reliability

The Cronbach's alpha value for the full Chinese Stigma Scale of Epilepsy was 0.936, and above 0.7 for each individual item. Cronbach's alpha was 0.887 for the factor 1, 0.737 for the factor 2, 0.897 for the Factor 3, 0.900 for the factor 4, and 0.920 for the factor 5, as shown in Table 4.

## Discussion

To validate the Chinese version of the SSE for accessing the epilepsy-related stigmatization of the public and to explore effective stigma reducing interventions, our study translated the SSE into Simplified Mandarin Chinese and qualified the translated scale with psychometric analysis, such as EFA, CFA, and internal consistency analysis. We provided clear evidence for the reliability and validity of the Chinese version of SSE.

Most of the participants included in our study were recruited *via* convenience sampling by randomly distributing questionnaires on the streets. Moreover, a part of the participants was enrolled through an online platform named Wenjuanxing, which enabled a wider coverage of our sample in geographical locations and life backgrounds. Indeed, the demographic data of our study indicated that our respondents had good social and cultural representation. The age of our respondents is mainly 18–60 years (95.4%). Compared with the study form Italy, our male-female ratio was more appropriate, avoiding the effect of excessive positive reactions of women on the results. As for the marital status and educational levels and medical background, there was no difference between the previous study and ours (38, 39). Additionally, most of our participants did not have family history of epilepsy or relatives with epilepsy (3.2%), whose proportion was lower than that of the previous study conducted in other counties (12.4%) (39).

With regard to reliability, the Cronbach's alpha value in our study was 0.936, and the Cronbach's alpha value of each factor of the five latent traits was above 0.7, indicating excellent internal consistency of our Chinese version of the SSE. Content validity is an important step before exploring the structure validity. It refers to the degree of agreement between the content actually measured by a scale and the content to be measured. The SSE was evaluated by experts and calculated CVI value above 0.78 indicated that the scale had good content validity.

For the structure of SSE, previous study has fitted SSE into a model with two latent traits using the exploratory item response theory (IRT) analysis. The first latent trait reflected the difficulties associated with epilepsy, whereas the second one reflected the emotions associated with epilepsy (40). Later, the Czech version was studied by Dana Brabcova et al. which extracted four-factor structure of SSE. The factor 1 and factor 3 were associated with responses of PWEs to epilepsy in their personal life and the effect of epilepsy on their study and work, whereas the factor 2 and factor 4 were associated with the emotional reaction of public to epilepsy, and their emotional perspective about PWEs (31). The Chinese version of SSE structure was a little different from others, we extracted a 6-factor structure of SSE by using the EFA. Factor 1 reflected the scare of respondents, and factor 2 reflected the sympathy of respondents, when they witnessed a seizure attack. The emotional reaction of the public to epilepsy was divided into two aspects, which might be contributed to cultural differences. Essentially, fear and sympathy are two different emotional reactions. Fear refers to a strong depressive emotional experience when people are facing a certain dangerous situation, trying to get rid of it but unable to do anything. Sympathy refers to a caring and understanding emotional response for the suffering and misfortune of others. Specifically, many people in our study said that “I was not scared when I witnessed an epileptic seizure, but I felt sad and pity for him/her.” The factor 3 reflected the difficulties faced by PWEs. The factor 4 reflected the thoughts of respondents about how PWEs felt, factor 5 reflected the prejudice associated with epilepsy, and the factor 6 reflected the knowledge of epilepsy. To be honest, the factor structure of Chinese version has been slightly modified from the original version, which might limit its application in other populations.

Items 1 and 13 were proved to be problematic and removed from the model. For item 1, it was the only item of factor 6, and thus incapable of being included in the model to evaluate the validity. Even though the phrase “be able to control their own epilepsy” sounds quite ambiguous when translated into Chinese, about 61.3% of our subjects believed that epilepsy could not be controlled by PWEs themselves or effective treatments. It is the Chinese people's subconscious understanding of epilepsy, thus forming an independent factor. Besides, the item 1 was not suitable for factor structure analysis just for statistical reasons, rather than incapacity of quantifying stigma. In fact, lack of knowledge is one aspect of stigma. People with a more knowledge or a higher education may have a lower score for stigma. In the case of item 13, it was removed for the reason that its factor loading in factor 3 and factor 4 were both higher than 0.5, which meant that item 13 had lower discriminant validity. In fact, the items 1 and 13 were also excluded in the recently validated Zambian and Czech version of the scale. They excluded items 1 and item 13 for their low factor load (31, 40).

We further validated the structural validity, convergent validity, and discriminant validity of the Chinese version of SSE. Structural validity refers to whether the structure of the scale is consistent with the theoretical assumptions of tabulation. The value of all metrics of the structural validity was excellent, indicating that the 5-factor construct of the scale fitted well. Convergent validity test showed that the item belonging to each factor was highly representative, and the discriminant validity test indicated the factors have a certain correlation, but they had a certain degree of discrimination between each other. Furthermore, we found that participants with medical background scored lower in factor 1 (the fear of seizure attacks) and factor 2 (sympathy for PWEs), which may be related to their deeper understanding of epilepsy. However, participants with family history of epilepsy scored higher on the total score and factor 2 (sympathy for PWEs), factor 3 (difficulties faced by PWEs), and factor 5 (discrimination against PWEs), which is in line with the fact we have observed clinically. Based on the above results, we hold the view that medical background and family history of epilepsy had an effect on the stigma, which is instructive for the application of the scale in the future and provides a theoretical basis for the design and grouping of subsequent studies on epilepsy stigma.

One limitation of the study is that a part of our respondents was recruited by an online platform, by which it may not include people who rarely access to computers or mobile phones, and those less educated people who were incapable of filling the scale online. Meanwhile, since our subjects mainly from Street random sampling and an online platform, we cannot carry out the test-retest reliability test. Besides, our study was conducted in Changsha, urban sampling could lower the participation rate of rural subjects thus affecting the results, as the incidence of stigma among PWEs in rural areas is higher than that of urban areas in China (13). Therefore, further studies with an adequate sample in different regions of China are needed.

Social stigma continues among PWEs. It brings them negative emotions such as anxiety and depression, restricts patients from seeking social support, and greatly affects the quality of life in PWEs. Therefore, it is crucial to formulate an effective public intervention for reducing stigma, starting with development and validation of an accurate Stigma Scale of Epilepsy, which might quantify the extent of stigma. Only when we are aware of the existence of epilepsy-associated stigma and psychosocial burden of PWEs can we have a deeper understanding of it, which may contribute to explore stigma reduction interventions to overcome prejudices, the false cognition, and to create a better social environment for PWEs and their family.

## Conclusions

This study has shown that the Chinese version of the SSE is a valid and reliable measurement instrument.

## Data Availability Statement

The raw data supporting the conclusions of this article will be made available by the authors, without undue reservation.

## Ethics Statement

The studies involving human participants were reviewed and approved by the Ethics Committee of the Xiangya Hospital of Central South University [No. 201912528]. Written informed consent to participate in this study was provided by the participants' legal guardian/next of kin.

## Author Contributions

YW and KH contributed to the acquisition of data, and the drafting of the manuscript for content and interpretation of data. LF and BX contributed to the study design and revision of manuscript. SW contributed to data analysis.

## Funding

This work was supported by the Natural Science Foundation of China [Grant Numbers: 82071461 and 81771407].

## Conflict of Interest

The authors declare that the research was conducted in the absence of any commercial or financial relationships that could be construed as a potential conflict of interest.

## Publisher's Note

All claims expressed in this article are solely those of the authors and do not necessarily represent those of their affiliated organizations, or those of the publisher, the editors and the reviewers. Any product that may be evaluated in this article, or claim that may be made by its manufacturer, is not guaranteed or endorsed by the publisher.
