# Supplementary material for: Validation of the Chinese Version of the Stigma Scale of Epilepsy
Source: Front Neurol. 2022 Feb 7;13:796296. doi: 10.3389/fneur.2022.796296 (PMC8858795; doi:10.3389/fneur.2022.796296)
Supplement: Supplementary file 1 [file Table_1.docx]

Supplementary table 1. Effect of Medical background and Family history of epilepsy on SSE score

|  | participants with medical background | participants without medical background | P-value of Mann- Whitney test | participants with Family history of epilepsy | participants without Family history of epilepsy | P-value of Mann- Whitney test |
| --- | --- | --- | --- | --- | --- | --- |
| factor 1 | 4.00(1.50) | 4.00(2.00) | **<0.001** | 4.00(3.50) | 4.00(3.00) | 0.666 |
| factor 2 | 6.00(3.00) | 7.00(3.00) | **0.002** | 8.00(1.00) | 6.00(3.00) | **0.029** |
| factor 3 | 18.00(10.00) | 19.00(9.50) | 0.7 | 24.50(8.75) | 19.00(9.00) | 0.052 |
| factor 4 | 17.00(6.50) | 19.00(7.00) | 0.169 | 24.00(8.50) | 18.00(6.00) | **0.019** |
| factor 5 | 13.00(5.00) | 12.00(5.00) | 0.689 | 17.00(6.75) | 12.00(5.00) | **0.015** |
| factor 6 | 1.00(1.00) | 1.00(1.00) | 0.93 | 1.00(1.00) | 1.00(1.00) | 0.598 |
| SSE score | 58.00(20.50) | 62.00(21.50) | 0.135 | 79.00(21.00) | 61.00(21.00) | **0.007** |
